# CFTR-regulated MAPK/NF-κB signaling in pulmonary inflammation in thermal inhalation injury

**DOI:** 10.1038/srep15946

**Published:** 2015-10-30

**Authors:** Zhi Wei Dong, Jing Chen, Ye Chun Ruan, Tao Zhou, Yu Chen, YaJie Chen, Lai Ling Tsang, Hsiao Chang Chan, Yi Zhi Peng

**Affiliations:** 1 State Key Laboratory of Trauma, Burns and Combined Injury, Chongqing Key Laboratory for Proteomics Disease, Institute of Burn Research, Southwest Hospital, the Third Military Medical University, Chongqing, China; 2 Epithelial Cell Biology Research Center, School of Biomedical Sciences, Faculty of Medicine, The Chinese University of Hong Kong, Hong Kong, People’s Republic of China

## Abstract

The mechanism underlying pulmonary inflammation in thermal inhalation injury remains elusive. Cystic fibrosis, also hallmarked with pulmonary inflammation, is caused by mutations in CFTR, the expression of which is temperature-sensitive. We investigated whether CFTR is involved in heat-induced pulmonary inflammation. We applied heat-treatment in 16HBE14o- cells with CFTR knockdown or overexpression and heat-inhalation in rats *in vivo.* Heat-treatment caused significant reduction in CFTR and, reciprocally, increase in COX-2 at early stages both *in vitro* and *in vivo*. Activation of ERK/JNK, NF-κB and COX-2/PGE_2_ were detected in heat-treated cells, which were mimicked by knockdown, and reversed by overexpression of CFTR or VX-809, a reported CFTR mutation corrector. JNK/ERK inhibition reversed heat-/CFTR-knockdown-induced NF-κB activation, whereas NF-κB inhibitor showed no effect on JNK/ERK. IL-8 was augmented by heat-treatment or CFTR-knockdown, which was abolished by inhibition of NF-κB, JNK/ERK or COX-2. Moreover, *in vitro* or *in vivo* treatment with curcumin, a natural phenolic compound, significantly enhanced CFTR expression and reversed the heat-induced increases in COX-2/PGE_2_/IL-8, neutrophil infiltration and tissue damage in the airway. These results have revealed a CFTR-regulated MAPK/NF-κB pathway leading to COX-2/PGE_2_/IL-8 activation in thermal inhalation injury, and demonstrated therapeutic potential of curcumin for alleviating heat-induced pulmonary inflammation.

Thermal inhalation injury is quite common in fire victims, especially in compartment fires, and is the leading cause of mortality in fire-related injuries^1^. One of the most notable pathophysiological characteristics of inhalation injury is overwhelming pulmonary inflammation^2^, which contributes to acute lung injury (ALI) or acute respiratory distress syndrome (ARDS). In inhalation injury, both heat and smoke cause inflammation to the respiratory tract. Current studies are mostly focused on the chemical damages to the lung attributed to smoke^3^, whereas damages caused by heat are thought to do little harm to the lower respiratory tract. A previous study on heat-induced inhalation injury suggests that pneumonia characterized by respiratory inflammation is the most significant and lethal complication^4^. However, whether heat, as the single factor, can induce pathophysiological changes, especially inflammatory responses of lower respiratory tract, remains an open question.

It has been reported that many crucial proinflammatory mediators, including COX-2/PGE_2_and IL-8, are involved in instigating and intensifying the pulmonary inflammatory cascade, contributing to ALI and ARDS^5^. It has been shown that COX-2 levels increase concomitantly with the severity of ALI, whereas COX-2 specific inhibitors attenuates proinflammatory cytokines, chemokines, and ALI in both burn-induced and other ALI animal models^6^,^7^, suggesting that despite of the complex network of inflammation and multiple actions of cytokines, COX-2/PGE_2_ might be a key mediator and thus a promising target in treating burn-related ALI. COX-2 is an inducible enzyme triggered by numerous stimuli, including cytokines, oxidants, mitogens, and microbial products^8^. COX-2 can induce a series of proinflammatory mediators, including IL-1β, IL-6 and IL-8^9^among which, IL-8, a chemokine for neutrophils, is well recognized to play a crucial role in airway inflammation^10^. It has been reported that COX-2 is regulated by mitogen-activated protein kinase (MAPK) and/or nuclear factor kappa-light-chain-enhancer of activated B cells (NF-κB) signalings in various types of cells and tissues, including airway cells^11^,^12^. Interestingly, upregulation of COX-2/PGE_2_ through ERK/NF-κB in a mouse model of severe burn-induced ALI has also been reported^6^. However, whether this proinflammatory signaling can be activated by heat alone during thermal inhalation injury is not known.

Cystic fibrosis (CF), a common autosomalrecessivedisorder caused by mutations of the gene encoding an anion channel, CFTR, is characterized by chronic airway inflammation with excessive production of inflammatory mediators, leading to exaggerated inflammatory response^13^, which resembles pulmonary inflammation after thermal inhalation injury. Both NF-κB and MAPK pathways have been implicated in mediating the excessive inflammatory responses of CF airway epithelia, especially in the induction of COX-2 and PGE_2_^11^,^14^,^15^. It has been reported that NF-κB is abnormally activated in CF airway epithelial cells^16^ and its activation has been shown to be dependent on CFTR trafficking and Cl^−^ channel function^16^. Our previous study has also demonstrated the involvement of a NF-κB-COX-2/PGE_2_ positive feedback loop, which is negatively regulated by CFTR under normal condition but augmented with defective CFTR, in the pathogenesis of CF airway inflammation^17^. Mutation of CFTR and loss of function of CFTR have also been shown to abnormally activate MAPKs^18^,^19^, leading to enhanced COX-2 transcription. Of note, functional expression of CFTR to the plasma membrane is known to be temperature sensitive and defective CFTR trafficking to the plasma membrane due to its mutation, DF508, is known to be rescued by lowering temperature^20^. Consistently, the expression of CFTR has been shown to be downregulated by heat in primary rat Sertoli cells^21^. Given the reported involvement of CFTR in regulating inflammatory responses in the airways, we hypothesized that thermal inhalation may induce downregulation of CFTR in bronchial epithelial cells leading to activation of MAPK and/or NF-κB pathways and excessive COX-2/PGE_2_, and thus, IL-8 production, contributing to exuberant airway inflammation seen in inhalation injury. We undertook the present study to test this hypothesis and to explore a treatment strategy targeting the CFTR-regulated signaling pathway.

## Results

### Time-dependent expression profile of CFTR and COX-2 after heat treatment *in vitro* and *in vivo*

**Figure 1:**
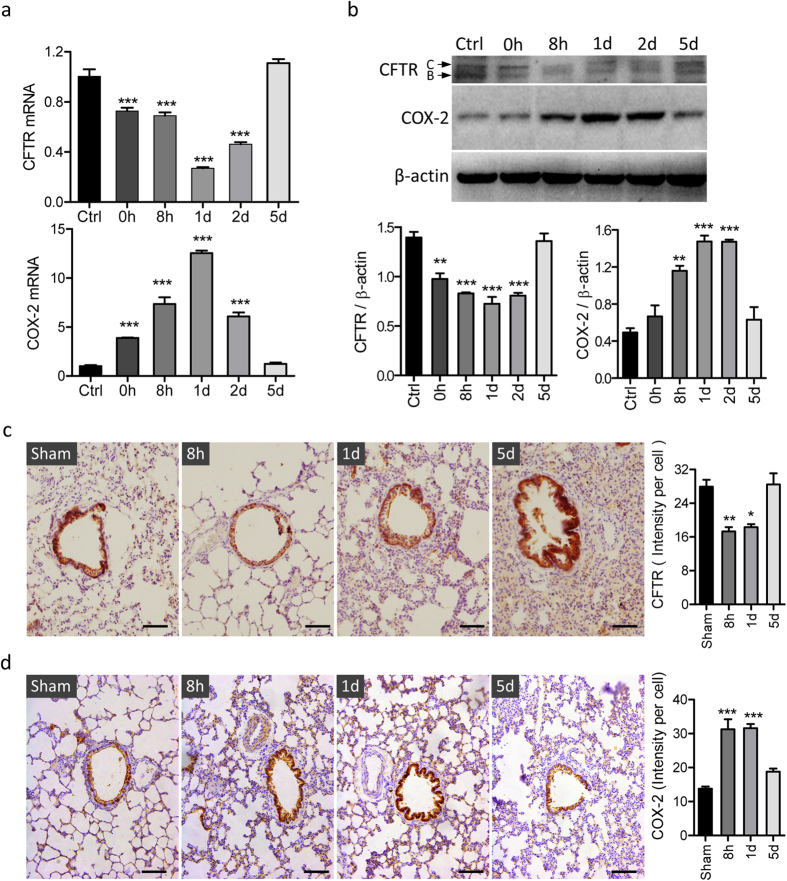
Heat-induced temporal changes in CFTR and COX-2 expression in airway epithelial cells *in vitro* and *in vivo*. (**a,b**) 16HBE14o- cells were cultured at 37 °C till 80% confluence before incubated at 52 °C for 5 min as heat-treatment and subsequently back at 37 °C for recovery. Cells were collected before (Ctrl), after heat-treatment (0 h), or after recovery for 8 hours (8 h) to 5 days (5d) for QRT-PCR (**a**) and western blot (**b**) analysis of CFTR and COX-2. Data are means ± SEM from at least three independent experiments. ***P < 0.001, **P < 0.01 as compared to Ctrl. n = 3. One-way ANOVA. (**c,d)** Representative images and corresponding semi-quantification of immunohistochemistry staining for CFTR (**c**) and COX-2 (**d**) in rat lung tissues 8 h-5d after inhalation of heated air. Same surgical procedures without the heat-inhalation were used in rats of the sham group. The intensity of CFTR and COX-2 positive signals was calibrated with the epithelial cell counts. ***P < 0.001, **P < 0.01 as compared to Ctrl. n = 3. One-way ANOVA. Bars = 100 μm (**c,d**).

We first examined whether heat could alter CFTR and COX-2 expression in 16HBE14o cells. QRT-PCR results showed a time-dependent significant reduction in CFTR mRNA after heat treatment, with the lowest level observed on day 1, which returned to normal level on day 5 after the treatment (Fig. 1a). Intriguingly, COX-2 mRNA expression levels exhibited a reciprocal pattern, with rapid significant increases after heat treatment, reaching a maximal level responding to the lowest level of CFTR observed 1 day after heat treatment, which returned to the basal level on day 5 (Fig. 1a). Also, the protein expression of CFTR and COX-2 exhibited a reciprocal pattern as detected by Western blot, in a similar time-dependent fashion (Fig. 1b). It is of note that both band C indicating mature CFTR and band B representing immature CFTR were downregulated after heat treatment. We also examined time-dependent changes in CFTR and COX-2 expression in a rat model of thermal inhalation injury. Heated air (~50 °C) produced by a heat gun was blown into the trachea to mimic the case in compartment fires. Immunohistochemistry (IHC) staining detected changes in CFTR and COX-2 expression levels after heat treatment, a significant reduction in CFTR expression between 8 h to 1 day corresponding to a significant increase in COX-2 in the same period of time. The recovery of CFTR expression on day 5 was accompanied by the returning of COX-2 to basal level (Fig. 1c,d). The reciprocal expression pattern of CFTR and COX-2 after heat treatment are consistent with that observed *in vitro*.

### Activation of MAPKs and NF-κB in airway epithelial cells by heat treatment

**Figure 2:**
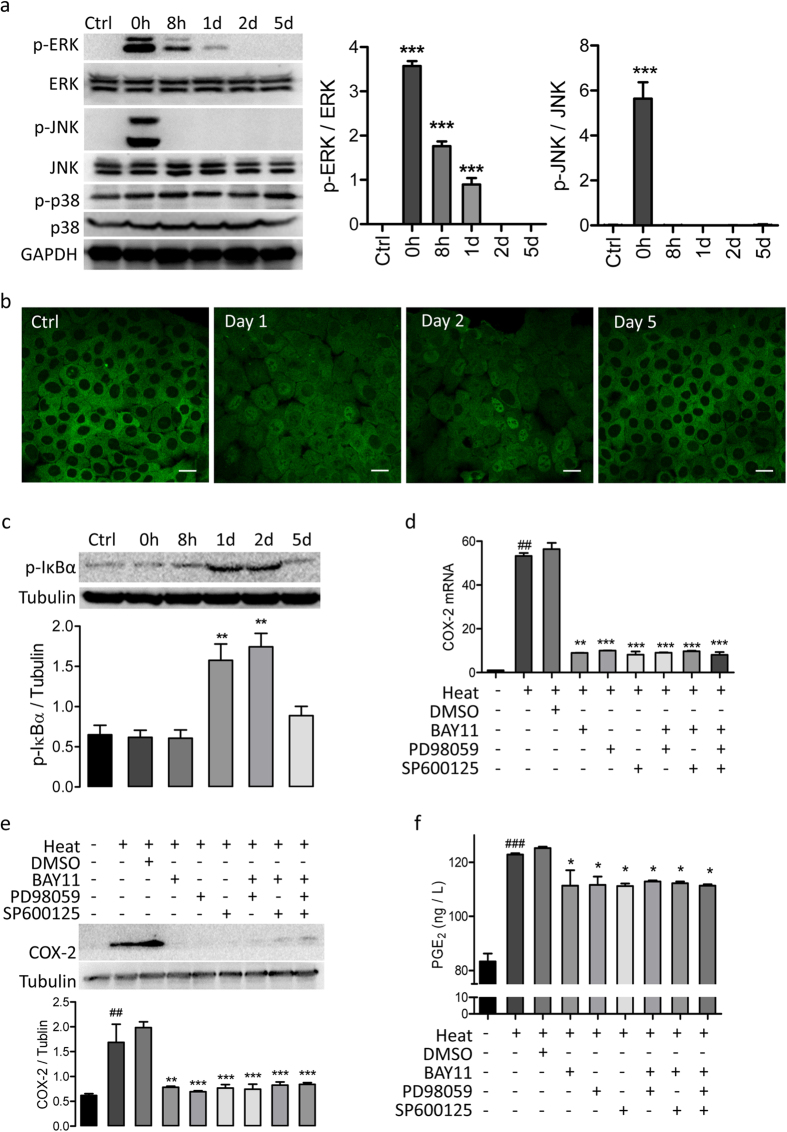
Heat-induced activation of MAPKs and NF-kB in 16HBE14o- cells. (**a**) Western blots *(Left)* and corresponding quantification *(Right)* for p-ERK, ERK, p-JNK and JNK in 16HBE14o- cells before (Ctrl) and 0 h-5d after the heat treatment. n = 3. ***p < 0.001. One-way ANOVA. (**b**) Representative images of immunostaining for NF-κB P65 in 16HBE14o- cells before (Ctrl) and after the heat treatment (Day 1–5) Bars = 100 μm. (**c**) Western blots *(Top)* and corresponding quantification (*Bottom*) for p-IκBα in 16HBE14o- cells before (Ctrl) and 0 h-5d after the heat treatment. (**d–f**) QRT-PCR (**d**) and western blot (**e**) analysis of COX-2, and ELISA detection of PGE_2_ production (**f**) in 16HBE14o- cells before (–) or 1d after (+) heat-treatment in the presence (+) or absence (–) of inhibitors of NF-κB (BAY11, 40 μM), ERK (PD98059, 20 μM), JNK (SP600125, 40 μM) or DMSO as vehicle control. ^###^P < 0.001, ^##^P < 0.01 as compared to cells without heat-treatment. ***P < 0.001, **P < 0.01, *P < 0.05 as compared to DMSO-treated cells. n = 3 (**d**–**f**).One-way ANOVA.

Since both MAPKs and NF-κB are known transcription factors controlling the downstream transcription of COX-2, we proceeded to test which of the two was or whether both were responsible for the upregulation of COX-2 expression observed after heat treatment in 16HBE14o- cells. Western blot results showed an immediate and significant phosphorylation of ERK and JNK, two MAPKs, upon heat treatment (Fig. 2a). No significant differences in phosphorylation of p38, another MAPK, was detected after heat treatment (Fig. 2a). After heat treatment, the translocation of p65, a NF-κB member, from the cytoplasm to nucleus was observed by immunofluorescence staining 1 day after heat treatment. p65 remained in the nucleus on day 2, and then returned to the cytoplasm on day 5 (Fig. 2b). Temporal change in the expression levels of the phosphorylated NF-κB inhibitor α (p-IκBα), which is an indicator of NF-κB activation, after heat treatment was detected by western blot with maximal levels observed on day 1 and 2 (Fig. 2c). Furthermore, both QRT-PCR and western blot analysis showed that the heat-induced increase in COX-2 expression, mRNA (Fig. 2d) or protein (Fig. 2e), could be reversed by NF-κB inhibitor (BAY11), ERK inhibitor (PD98059) or JNK inhibitor (SP600125) alone. Combination of NF-κB and MAPKs inhibitors did not further decrease COX-2 expression (Fig. 2d,e). Increase in PGE_2_ levels, as detected by ELISA, was also induced by heat treatment, which could be reversed by either one of the three inhibitors alone with no additive effect if added together (Fig. 2f). However, BAY11, PD98059 or SP600125 could not recover heat-induced loss of CFTR expression (as shown in the supplementary Fig. S1, online).

### Involvement of CFTR in mediating the heat-induced activation of MAPK/NF-κB/COX-2/PGE_2_ signaling pathway *in vitro*

**Figure 3:**
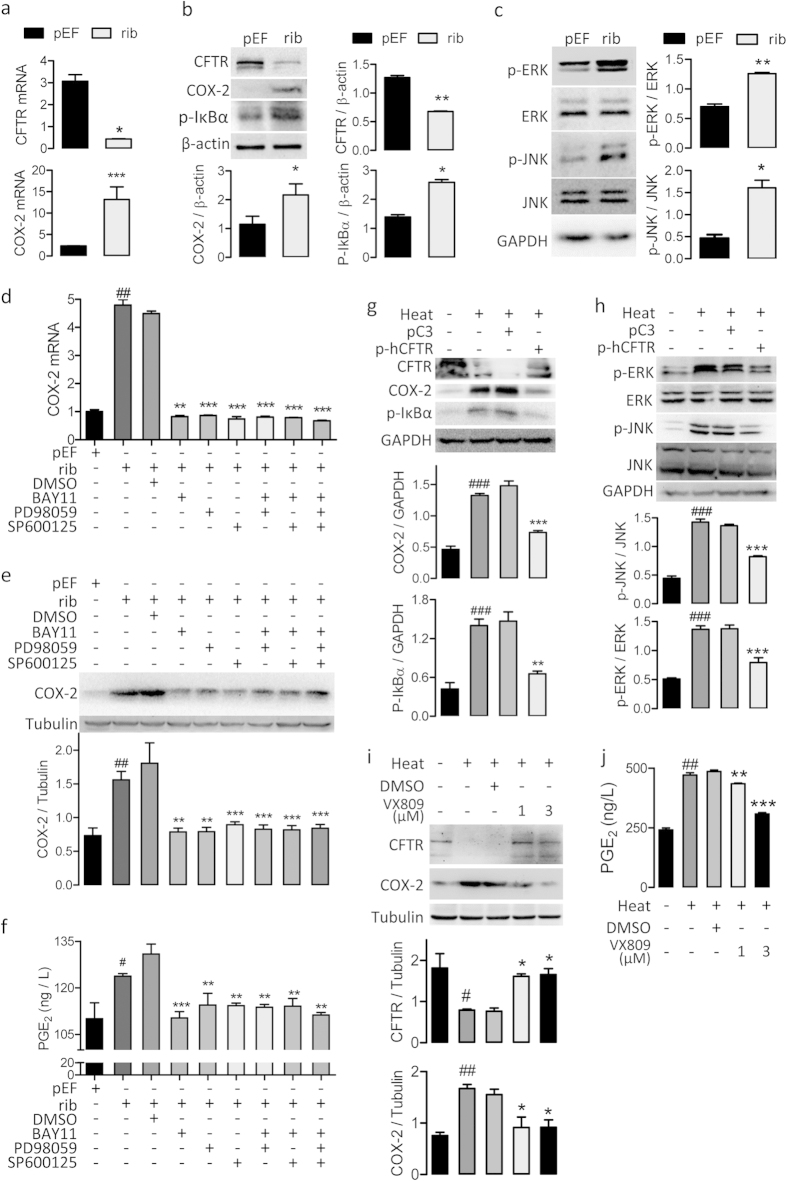
CFTR mediates heat-induced activation of MAPK/NF-κB/COX-2/PGE_2_ in 16HBE14o- cells. (**a**) QRT-PCR analysis of CFTR and COX-2 in cells transfected with CFTR-silencing ribosome vectors (rib) or empty pEF6/V5-His vectors as negative control (pEF). ***P < 0.001, *P < 0.05. n = 3. t-test. (**b–c**) Western blotting for CFTR, COX-2 and p-IκBα (**b**), and p-ERK, ERK, p-JNK and JNK (**c**) in cells transfected with rib or pEF. β-actin or GAPDH was used as loading control. **P < 0.01, *P < 0.05. n = 3. t-test. (**d–f**) QRT-PCR (**d**) and western blot (**e**) analysis of COX-2, and ELISA detection of PGE_2_ production (**f**) in cells with (+) or without (−) the transfection of pEF/rib in the presence (+) or absence (−) of BAY11(40 μM), PD98059 (20 μM), SP600125 (40 μM) or DMSO as vehicle control. Tubulin was used as loading control. ^##^P < 0.01, ^#^P < 0.05 as compared to pEF transfected cells. ***P < 0.001, **P < 0.01 as compared to DMSO-treated cells. n = 3 (D-F).One-way ANOVA. (**g–h**) Western blotting for CFTR, COX-2 and p-IκBα (**g**), and p-ERK, ERK, p-JNK and JNK (**h**) before (−) and after (+) heat-treatment with (+) or without (−) the transfection of pEGFPC3 vector conjugated with human CFTR (p-hCFTR) or empty pEGFPC3 vectors (pC3).GAPDH was used as loading control. ^###^P < 0.001 as compared to cells without heat-treatment. ***P < 0.001, as compared to pC3-transfected cells. (**i,j**) Western blotting for CFTR and COX-2 (**i**), and ELISA for PGE_2_ and IL-8 (**j**) in cells before (−) and after (+) heat-treatment with (+) or without (−) the treatment withVX-809 (1–3 μM). Tubulin was used as loading control. ^#^P < 0.05; ^##^P < 0.01, ^###^P < 0.001 as compared to cells without heat-treatment. *P < 0.05, **P<0.01, ***P < 0.001as compared to heat-treated cellstreated with DMSO as vehicle control.

If heat-induced downregulation of CFTR is the key event leading to the activation of MAPKs/NF-κB, knocking down of CFTR should mimic the effect of heat treatment. We tested this by knocking down CFTR in 16HBE14o cells, which led to a significant increase of COX-2 mRNA and protein expression concomitant with phosphorylation of IκBα, ERK and JNK (Fig. 3a–c).Furthermore, the CFTR knockdown-induced increases in COX-2 or PGE_2_ were significantly reduced by pretreatment with BAY11, PD98059 or SP600125 alone, with no additive effect when treated in combination (Fig. 3d–f). Moreover, successful overexpression of CFTR by transient transfection of vectors encoding human CFTR (as shown in supplementary Fig. S2, online) significantly reduced the heat-increased expression of p-IκBα, p-ERK1/2, p-JNK and COX-2 (Fig. 3g,h). In addition, VX-809, the reported corrector for the CFTR mutation, reversed the heat-induced effects in cells including reduction in CFTR, upregulation of COX-2 and increases in PGE_2_ (Fig. 3I,j).

### MAPK is the upstream of NF-κB in the heat-induced response

**Figure 4:**
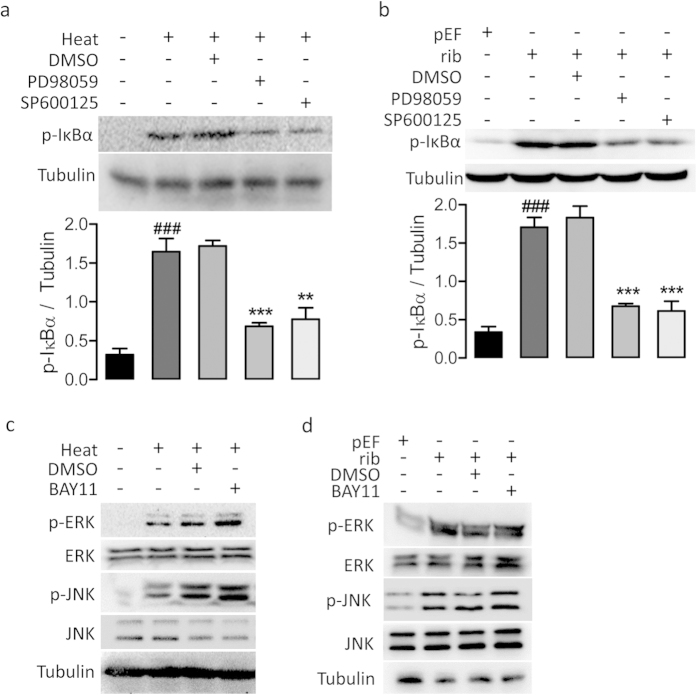
MAPKs activation precedes NF-κB signaling in response to heat or CFTR knockdown in 16HBE14o- cells. (**a,b**) Western blotting for p-IκBα in cells with (+) or without (−) heat treatment (**a**), transfection of pEF/rib (**b**) in the presence (+) or absence (−) of PD98059 (20 μM), SP600125 (40 μM) or DMSO as vehicle control. Tubulin was used as loading controls. ^###^P < 0.001 as compared to cells without heat-treatment (**a**), or transfected with pEF (**b**). ***P < 0.001, **P < 0.01 as compared to DMSO-treated cells. n = 3, One-way ANOVA. (**c–d**) Western blotting for p-ERK, ERK, p-ERK and JNK in cells with (+) or without (−) heat-treatment (**c**) or transfection of pEFor rib (**d**) in the presence (+) or absence (−) of BAY11 (40 μM)or DMSO as vehicle control. Tubulin was used as loading controls. No visible effect of BAY11 was detected.

Since the above experiments seemed to indicate that MAPK and NF-κB are involved in the same signaling pathway, we proceeded to test the sequence of MAPK and NF-κB activation. Western blot analysis demonstrated that PD98059 or SP600125 could substantially block the NF-κB activation as shown by significantly reducing the heat- or CFTR knockdown-induced p-IκBα increases (Fig. 4a,b). However, NF-κB inhibitor BAY11 failed to produce significant effect on the heat- or CFTR knockdown-induced phosphorylation of either ERK or JNK (Fig. 4c,d). These results suggest that MAPK is the upstream of NF-κB in the heat-induced response.

### Heat-induced activation of CFTR/MAPK/NF-κB/COX-2/PGE_2_ pathway leads to increase in IL-8 production

**Figure 5:**
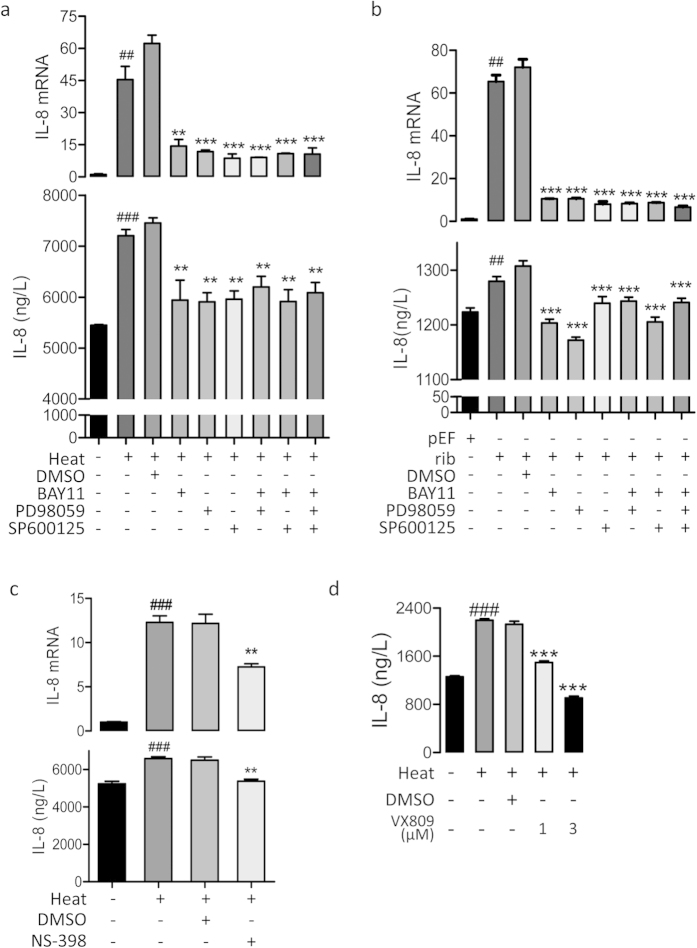
IL-8 production as a result of MAPK/NF-κB/COX-2/PGE_2_ activation by heat or CFTR knockdown in 16HBE14o- cells. (**a,b**) QRT-PCR analysis and ELISA detection of IL-8 production in cells with (+) or without (−) heat-treatment (**a**), transfection of pEF/rib (**b**) in the presence (+) or absence (−) of BAY11 (40 μM), PD98059 (20 μM), SP600125 (40 μM) or DMSO as vehicle control. ^###^P<0.001, ^##^P<0.01 as compared to cells without heat-treatment (**a**) or transfected with pEF (**b**). ***P<0.001, **P<0.01 as compared to DMSO-treated cells. n = 3, One-way ANOVA. (**c**) QRT-PCR analysis and ELISA detection of IL-8 in cells before (−) and after (+) heat-treatment in the presence (+) or absence (−) of the specific COX-2 inhibitor,NS-398 (10 μM) or DMSO as vehicle control. (**d**) ELISA detection of IL-8 in cells with or without treatment of VX-809.

We further examined whether the heat-activated CFTR/MAPK/NF-κB/COX-2/PGE_2_ pathway could lead to the production of the proinflammatory factor, IL-8. QRT-PCR results revealed thatIL-8 mRNA expression was increased by heat treatment (Fig. 5a) or CFTR knockdown (Fig. 5b) in 16HBE14o cells, which could be reversed by BAY11, PD98059 or SP600125 alone, with no additive effect if treated in combination. Similar results were obtained using ELISA measuring IL-8 in the culture media 1d after heat treatment or CFTR knockdown (Fig. 5a,b). Moreover, both QRT-PCR and ELISA data showed that the increase of IL-8 induced by heat treatment could be significantly reduced by a specific COX-2 inhibitor-NS-398 (Fig. 5c), suggesting the involvement of COX-2 in heat-induced IL-8 production. In addition, treatment with the CFTR mutation corrector, VX-809, also reversed the heat-induced increase in IL-8 (Fig. 5d).

### Curcumin mitigates heat-induced inflammation by upregulating CFTR in airway epithelial cells *in vitro* and *in vivo*

**Figure 6:**
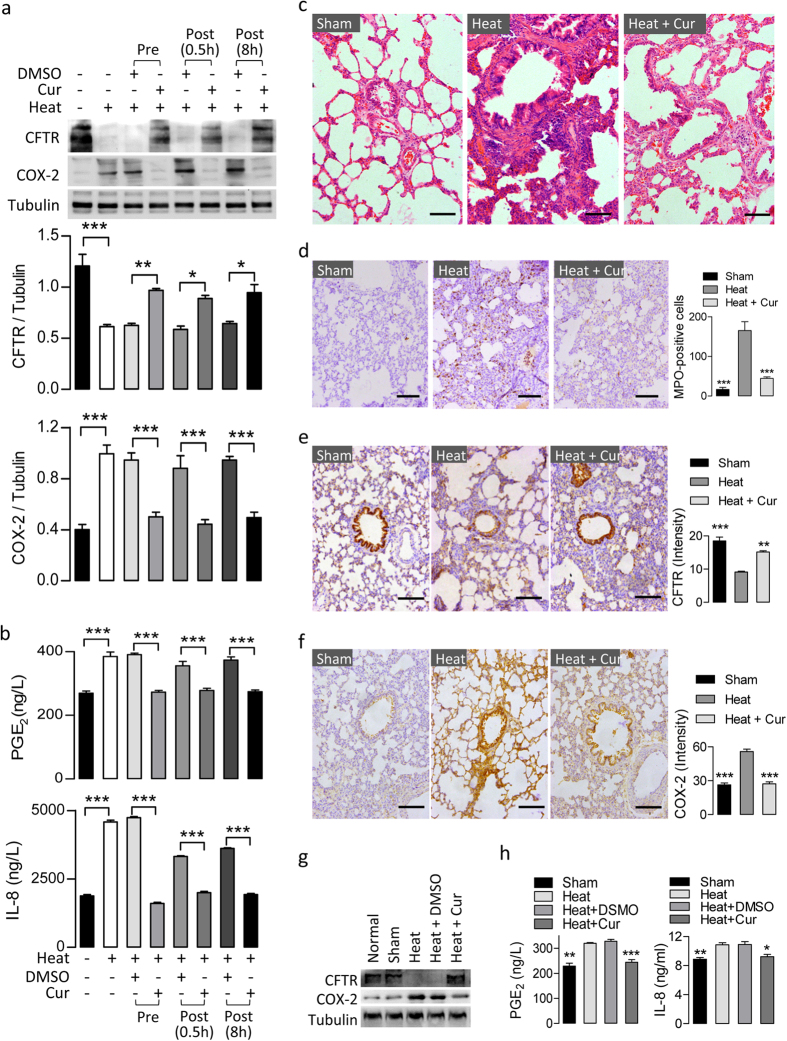
Curcumin mitigates heat-induced inflammation by upregulating CFTR in airway epithelial cells *in vitro* and *in vivo*. (**a,b**) Western blotting for CFTR and COX-2 (**a**), and ELISA detection of PGE_2_and IL-8 (**b**) in 16HBE14o- cells before (−) and after (+) heat-treatment in the presence (+) or absence (−) of curcumin (Cur, 10 μM)or DMSO as vehicle control. Curcumin was administrated either 4 hours before (Pre), 0.5 hour after (Post, 0.5 h), or 8 hours after (Post, 8 h) the heat treatment. Tubulin was used as loading control. *P < 0.05; **P < 0.01; ***P < 0.001. n = 3, One-way ANOVA. (**c**) Curcumin (Cur,10 mg/kg body weight) was nasally administrated in rats in addition to heat-inhalation. H&E images show airway morphology of rats with heat-inhalation alone (Heat), treated with Cur after heat-inhalation (Heat + Cur) or without heat-inhalation (Sham). (**d**) Immunohistochemistry staining for MPO in airway tissues from rats of the three groups. The number of MPO-positive cells in each group was counted and is shown as mean ± SEM. ***P < 0.001 as compared to rats with heat-inhalation alone. n = 3, One-way ANOVA. (**e,f)**Representative images and corresponding semi-quantification of immunohistochemistry staining for CFTR (**e**) and COX-2 (**f**) in airway tissues from rats of the three groups. The intensity of CFTR and COX-2 signal was calibrated with the epithelial cell counts. **P<0.01; ***P<0.001 as compared to rats with heat-inhalation alone. n = 3, One-way ANOVA. (**g**) Western blotting for CFTR and COX-2 in normal rat airway tissues (Normal) or tissues with (Heat) or without (Sham) heat-inhalation administrated with curcumin (Cur, 10 mg/kg body weight) or DMSO. (**h**) ELISA for rat PGE_2_ and IL-8 using homogenates of rat airway tissues with (Heat) or without (Sham) heat-inhalation administrated with curcumin (Cur, 10 mg/kg body weight) or DMSO Bars = 100μm.

Curcumin is a plant-derived compound shown to activate CFTR channels^22^, inhibit MAPK and NF-κB activaton^23^, and inhibit COX-2^24^, the key components of the heat-induced signaling examined in this study. We proceeded to test the effects of curcumin on airway epithelial cells under heat-treatment. Addition of curcumin (10 μM) 4 hours before, 0.5 hours after or 8 hours after the heat-treatment reversed the heat-induced downregulation of CFTR and increases in COX-2 (Fig. 6a), determined by western blot, PGE_2_ and IL-8 (Fig. 6b), determined by ELISA. We next tested possible effects of curcumin on the thermal inhalation injury rat model *in vivo*. Severe edema, interstitial inflammation and inflammatory cell infiltration were observed 2 days after inhalation injury as observed by H&E staining (Fig. 6c). Curcumin (10 mg/kg body weight/day, intranasal injection) strikingly decreased the degree of inflammation in the lung as indicated by H&E staining (Fig. 6c) and immunohistochemical staining of MPO, a marker of neutrophil infiltration and an indicator of acute lung injury, showing significant reduction in MPO-positive staining cell numbers in curcumin-treated group compared to the heat-injured group, with levels comparable to the sham group (Fig. 6d). Concomitantly, immunostaining (Fig. 6e,f), western blot (Fig. 6g) and ELISA (Fig. 6h) showed that after treating the heat-injured rats with curcumin, the expression of CFTR was increased and COX-2/PGE_2_/IL-8 were reduced significantly in the lung as compared to the heat-injured group without curcumin treatment, which were recovered to levels comparable to the sham or normal (no treatment) groups, suggesting that the anti-inflammatory effect of curcumin was mediated, at least in part, by elevating CFTR expression.

## Discussion

To the best of our knowledge, the present study is the first to investigate the possible involvement of heat, as an important contributing factor, in inducing inflammatory response in bronchial epithelial cells and has demonstrated the underlying signaling mechanism with CFTR as the key player.

This study has demonstrated that heat alone results in activation of COX-2 and production of IL-8, involving MAPKs as upstream event and NF-κB downstream. The time course studies, both *in vitro* and *in vivo* show a significant increase in COX-2 expression peaking at one day after the heat treatment, followed by a decrease back to normal level on day 5 post the heat treatment.The increases in COX-2, PGE_2_ and IL-8 production induced by heat-treatment is mediated by both MAPKs (JNK and ERK) and NF-κBsignalings since MAPKs (JNK and ERK) or NF-κB inhibitors could reverse the COX-2, PGE_2_ and IL-8 increase. IL-8 elevation induced by heat can also be reversed by a specific COX-2 inhibitor, suggesting IL-8 is downstream of COX-2. In this study, we identified that MAPKs (JNK and ERK) are upstream of NF-κB in the heat-induced response, since ERK or JNK inhibitor could abrogate the elevation of p-IκBα induced by heat, whereas NF-κB inhibitor showed no effect on ERK or JNK activation. Activation of MAPKs is detected sooner than activation of NF-κB after heat-treatment by western blotting, supporting the notion that MAPKs are upstream of NF-κB. Furthermore, the heat-induced COX-2 increase can be reversed by MAPKs or NF-κB inhibitor alone, with no additive effect if treated in combination, suggesting that MAPKs and NF-κB are on the same pathway.

The key finding of the present study is the involvement of CFTR in mediating the heat-induced signaling events and inflammatory response. Firstly, heat-treatment induces temporal changes of CFTR expression, which exhibits a reciprocal pattern of time-dependent COX-2 expression post heat treatment *in vitro* and *in vivo*. The change in either CFTR or COX-2 seems to be fast with no temporal gap between mRNA and protein, probably due to both quick synthetic kinetics and heat-caused instability of the two proteins^25^,^26^.It has been reported that airway inflammation peaks during the first 72 hours after inhalation injury^27^, which is coincides with the time frame of CFTR and COX-2 expression changes in the present study, suggesting that CFTR and COX-2 may play a significant role in the early pulmonary inflammatory response in inhalation injury. Secondly, knock down of CFTR mimics the proinflammatory effect of heat treatment, which can be offset by MAPKs (JNK and ERK) or NF-κB inhibitors as well, whereas overexpression of CFTR could dramatically attenuate the heat-induced responses. Of note, the CFTR expression level in the overexpression group is also reduced by the heat treatment (Supplementary Fig. S3, online); however this level of CFTR is comparable to the endogenous CFTR level before heat-treatment. Consistently, the heat-induced proinflammatory responses in the overexpression group were reversed to levels similar to the basal levels. Importantly, VX-809, a known CFTR mutation corrector, not only restored CFTR expression in these cells, but also prevented the proinflmmatory effects after the heat treatment, confirming the involvement of CFTR in the heat-induced inflammation in airway epithelial cells. Moreover, curcumin is found to effectively decrease the airway inflammation, with concomitant increase in CFTR expression levels *in vitro* and *in vivo*, suggesting that the anti-inflammatory effect of curcumin could be attributed by its effect on upregulation of CFTR. Of note, curcumin has been reported to potentiate CFTR channel function^28^,^29^. The activation of NFκB in CF has also been associated with the loss of CFTR channel function (e.g. Cl^−^/HCO_3_^−^ transport) of CFTR^16^,^30^. We also showed previously that CFTR inhibitor induced NFκB activation^16^, consistent with the association of CFTR channel function with NFκB activation. However, in a previous study, CHO cells transfected with G551D, a channel function-loss mutation of CFTR, did not affect NFκB activity, similarly to that observed in wtCFTR-transfected cells^17^. Interestingly, in the same study, a mutation in the channel function-regulating domain (R domain) of CFTR increased NFκB activity^17^. Thus, whether the heat-induced proinflammatory effects are associated with CFTR channel function loss and whether curcumin prevents heat-induced proinflammatory effects by potentiating CFTR channel function, await further investigation.

The present study has clearly demonstrated that heat-induced CFTR downregulation is responsible for the heat-induced inflammatory response. Lukacs *et al.* have demonstrated that the processing of CFTR protein in endoplasmic reticulum and its trafficking to the apical membrane are temperature dependent, as elevated temperature results in reduced CFTR expression on the cell membrane^26^. In the present study, we also observed decrease in both bands of CFTR proteins, the mature form in the membrane and the immature form in the cytoplasm, after heat treatment, as detected by Western blot and immunohistochemical staining, which are consistent with our previously observed heat-induced downregulation of CFTR in Sertoli cells from a cryptorchidism model^21^. Interestingly, in addition to temperature-sensitive alteration in protein trafficking of CFTR, we also detected alteration in the transcription of CFTR after heat treatment with a significant decrease in CFTR mRNA expression in bronchial epithelial cells although the exact mechanism remains to be elucidated. The temperature-sensitive regulation of CFTR may have physiological significance other than the case of thermal inhalation injury. Possible change of CFTR expression in the lung under high fever may induce or aggravate pneumonia. In addition to heat, CFTR expression has also been shown to be downregulated by a number of conditions, such as smoking^31^, which is also associated with pulmonary inflammation^32^. Taken together, the presently demonstrated involvement of CFTR downregulation in heat-induced inflammation may have broad implication in a wide range of lung inflammatory responses, similar to the exuberant lung inflammation seen in CF due to CFTR mutations^13^.

The present finding that CFTR is an upstream regulator of MAPKs/NF-κB, COX-2/PGE_2_ and IL-8 suggests that CFTR may hold great promise as a therapeutic target, as demonstrated in the present study with curcumin treatment. A large number of studies have illustrated the effects of curcumin on inhibiting MAPKs and NF-κB activation and COX-2 expression^23^,^24^,^33^, however, the underlying mechanism remains obscure. The presently observed upregulation of CFTR upon curcumin treatment *in vitro* and *in vivo* is consistent with the reports from others^22^,^34^. The present study thus provides a new explanation to the anti-inflammation effect of curcumin. Interestingly, in the present study, administration of curcumin to airway epithelial cells either pre- or post- heat treatment can elevate CFTR expression and subsequently reduce COX-2/PGE_2_/IL-8 levels. The observation that curcumin is effective in increasing CFTR expression and suppressing inflammation even 8 hours after heat treatment indicates a wide therapeutic window for clinical application in burn patients with inhalational injury. Of note, curcumin has been found to down regulate tobacco-induced NF-κB activation and COX-2 expression in human oral premalignant and cancer cells^33^.This effect of curcumin, in light of the present finding, is very likely through upregulating CFTR expression as well. Steroids, which are well known for their effect on suppressing inflammation and widely used for relieving lung inflammation in respiratory diseases, such as asthma^35^ and chronic obstructive pulmonary disease (COPD)^36^, may also exert their effect through upregulation of CFTR since a number of hormones, such as estrogen^37^ and glucocorticoid^38^, have been shown to have effect on CFTR expression. The presently demonstrated therapeutic effect of curcumin in heat-induced inflammation warrants future investigation exploring treatment strategy targeting CFTR for alleviating lung inflammation induced by different conditions other than thermal inhalation injury.

## Methods

### Animals and thermal inhalation injury

Adult male Sprague-Dawley rats were used to induce thermal inhalation injury. All procedures were approved by the Animal Ethical Committee of the Third Military Medical University and were carried out in accordance with the approved guidelines. The thermal inhalation injury was modified from a previously described method on dogs^39^. Adult male Sprague-Dawley rats were generally anesthetized by constant inhalation of isoflurane (5%) via a face mask. Tracheotomy was performed under anesthesia and a 1 cm catheter was inserted into the trachea, through which preheated dry air was blown into the airway by a heat-gun (TAK-3316E, Shenzhen Takgiko Techonology, Shenzhen, China) at a flow rate of 190 L/min for a total of 10 min with 1 min intervals in between each minute of heat inhalation. The air was preheated till 110 °C inside the heat-gun and tested to be around 50 °C when blown to the end of the catheter. Afterwards, rats were closed by sutures. Acetylpromazine (0.75 mg/kg body weight) was intraperitoneally injected into the rats to relieve the pain. The same procedure without heat inhalation was performed in the rats of the sham group. In some of the rats, cucurmin (10 mg/kg body weight) was intranasally injected 30 min before the heat treatment and daily administrated in the same way after the surgery.

### Cell culture

16HBE14o- cells were grown in minimum essential medium with 10% fetal bovine serum in CO_2_ incubator at 37 °C. To induce heat treatment, cells were incubated at 52 °C for 5 minutes and subsequently recovered at 37 °C. BAY11 (40 μM, Cat#B5556, Sigma, ST. Louis, USA), PD98059 (20 μM, Cat#P215, Sigma), SP600125 (40 μM, Cat#S5567, Sigma) or VX-809 (1 -3 μM, Cat#HY-13262, MCE) were added alone or in combination 4 hours before the heat treatment. Curcumin (10 μM, Cat#A600346-0005, Sangon Biotech, Shanghai, China) was administrated 4 hours before, 0.5 hour after or 8 hours after the heat treatment.

### CFTR knock-down and overexpression

To knock down CFTR, hammerhead ribozymes (pEF6/V5-rib, stated as rib in the following experiments) targeting at a specific GUC or AUC site to degrade CFTR mRNA were used as described previously^40^. CFTR was overexpressed using pEGFPC3 plasmid expressing wild-type human CFTR (p-hCFTR, a gift from Professor Tzyh-Chang Hwang, University of Missouri-Columbia, USA). Cells were seeded in six-well plates and grown till 70–80% before transfected with 4 μg empty pEF6/V5-His vectors (pEF, Invitrogen, Carlsbad, CA, USA), pEF vectors conjugated with the CFTR-targeting ribozymes (rib), empty pEGFPC3 vectors (pC3) or p-hCFTR using lipofectamine 2000 reagent (Invitrogen). 6 hours later, the medium was replaced with fresh complete growth medium with or without the inhibitors BAY11 (40 μM), PD98059 (20 μM) or SP600125 (40 μM). Cells were harvested 72 hours after the transfection.

### Quantitative real-time RT-PCR (QRT-PCR)

QRT-PCR was carried out using a cDNA Synthesis Kit (Cat#K1622, Thermo Scientific, Fremont, USA) and the SGExcel UltraSYBR Mixture (Cat#SK2956A, Sangon Biotech, Shanghai, China). The sequences of primers used were as follows: CFTR: TGCCCTTCGGCGATGTTT (forward), GCGATAGAGCGTTC CTCCTTG (reverse); COX-2: TGTGTTGACATCCAGATCAC (forward), ACATCATGTTTGAGCCCTGG (reverse); β-actin: CATGTACGTTGCTATCCAGGC (forward), CTCCTTAATGTCACGCACGAT (reverse); IL-8: GGTGGAGTTTGCCAAGGAG (forward), TTCCTTGGGGTCCAGACAGA (reverse).

### Western blot

Western blotting was carried out as previously described^16^. Antibodies used in this study were listed as follows: CFTR (1:200, Cat#ACL-006, Alomone labs, Jerusalem, Israel), COX-2 (1:200, Cat#160106, Cayman chemical, Ann Arbor, USA), P-JNK (1:1000, Cat#9255, Cell Signaling Technology, Boston, USA), JNK (1:1000, Cat#9252, Cell Signaling Technology), P-Erk1/2 (1:1000, Cat#4370, Cell Signaling Technology), Erk1/2 (1:1000, Cat#4695, Cell Signaling Technology), P-IκBα (1:500, Cat#AB55066, Sangon Biotech), GAPDH (1:5000, Cat#KC-5G5, Kangcheng Biotech, Shanghai, China), Tubulin (1:500, Cat#10759-1-AP, Proteintech, Chicago, USA) and β-actin (1:1000, Cat#60008-1-lg, Proteintech).

### Immunohistochemistry

Tissues were fixed in 4% paraformaldehyde and paraffin-embedded. Sections at the same depth of the airway with similar degree of heat exposure were used for comparison between different groups. GT Vision III IHC kit (Cat#GK500710, Gene Company, Hong Kong, China) was used with antibodies against CFTR (1:80, Cat#AB60166a, Sangon Biotech), COX-2 (1:50, Cat# 160106, Cayman chemical) and myeloperoxidase (MPO, Cat#RB373-R7, Thermo scientific). Hematoxylin was used for counterstaining. Pictures were taken by Leica Microsystems (DFC300FX, Leica, Switzerland) and quantified by Image pro-Plus. Five random fields were examined per animal to determine the number of cells staining positive for MPO. Five random fields were examined per animal to detect CFTR and COX-2 expression level. The intensity of CFTR and COX-2 were calibrated with the epithelial cell counts.

### Immunofluorescence staining

Cells grown on coverslips were fixed with acetone, blocked in 10% goat serum in PBS and incubated with antibody against NF-κB P65 (1:50, Cat#8242, Cell Signaling Technology) at 4 °C overnight, followed by 1 hour incubation with Fluorescein (FITC)-conjugated AffiniPure Goat Anti-Rabbit IgG(H+L) (1:25, Cat#111-095-045, Jackson Immuno Research, PA, USA) at room temperature. Pictures were taken by a laser scanning confocal microscope (LSM 780, Carl Zeiss, Germany)

### ELISA

ELISA kits for human PGE_2_ (ESK5090-96 T) and IL-8 (ESK5204-96 T), and rat PGE_2_ (C547426-0096) and IL-8 (C506029-0096) were purchased from Sangon Biotech and used according to manufacturer’s instructions.

### Statistical analysis

Data are the mean ± sem. Students’-test was used when comparing two groups of variables. One-way ANOVA followed by Turkey post-test was used when there were more than two groups. P < 0.05 was considered to be statistically significant.

## Additional Information

**How to cite this article**: Dong, Z. W. *et al.* CFTR-regulated MAPK/NF-κB signaling in pulmonary inflammation in thermal inhalation injury. *Sci. Rep.* **5**, 15946; doi: 10.1038/srep15946 (2015).

## Supplementary Material

Supplementary Information
