# Supplementary material for: CFTR-regulated MAPK/NF-κB signaling in pulmonary inflammation in thermal inhalation injury
Source: Sci Rep. 2015 Oct 30;5:15946. doi: 10.1038/srep15946 (PMC4626762; doi:10.1038/srep15946)

CFTR-regulated MAPK / NF-κB signaling in airway inflammation induced by thermal inhalation injury

Zhi Wei Dong[1①], Jing Chen[1①], Ye Chun Ruan[2], Tao Zhou[1], Yu Chen[1], YaJie Chen[1], Lai Ling Tsang[2], Hsiao Chang Chan[2*], Yi Zhi Peng[1*]

1State Key Laboratory of Trauma, Burns and Combined Injury, Chongqing Key Laboratory for Proteomics Disease, Institute of Burn Research, Southwest Hospital, the Third Military Medical University, Chongqing, China

2 Epithelial Cell Biology Research Center, School of Biomedical Sciences, Faculty of Medicine, The Chinese University of Hong Kong, Hong Kong, People's Republic of China

[①]These authors contributed equally to this work.

*E-mail: hsiaocchan@cuhk.edu.hk； yizhipen@sina.com

Supplementary figures

Supple.Fig.S1. Effect of inhibition of MAPK or NFkB on CFTR expression in 16HBE14o-cells. Western blotting for CFTR in cells with (+) or without (-) heat-treatment in the presence (+) or absence (-) of BAY11, PD98059, SP600125 or DMSO as vehicle control. Tubulin was used as loading controls.

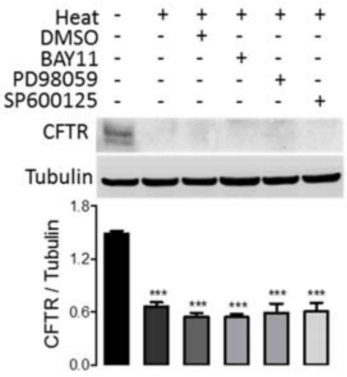

Supple.Fig.S2. Overexpression of CFTR in 16HBE14o- cells.

Western blotting for CFTR in cells transfected with pC3 (control vector) or p-hCFTR vector. β-actin was used as loading control.

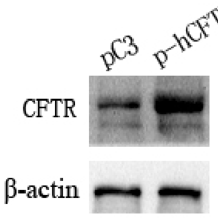

Supple.Fig.S3. Effect of heat on the expression of CFTR, p-IkBa and COX-2 in 16HBE14o- cells with CFTR overexpression. Western blotting for CFTR, p-IkBa and COX-2 in 16HBE14o- cells transfected with pC3 (control vector) or p-hCFTR vector treated with (+) or without (-) heat-treatment. β-actin was used as loading control.

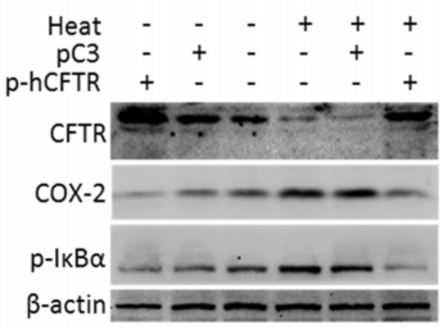

Supplement: Supplementary Information [file srep15946-s1.pdf]
